# A modified Hodgkin–Huxley model to show the effect of motor cortex stimulation on the trigeminal neuralgia network

**DOI:** 10.1186/s13408-019-0072-5

**Published:** 2019-05-31

**Authors:** Mohammadreza Khodashenas, Golnaz Baghdadi, Farzad Towhidkhah

**Affiliations:** Department of Biomedical Engineering, Amirkabir University of Technology, Tehran, Iran

**Keywords:** Computational modeling, Pain network, Neuropathic pain, Transcranial direct current stimulation

## Abstract

**Background:**

Trigeminal neuralgia (TN) is a severe neuropathic pain, which has an electric shock-like characteristic. There are some common treatments for this pain such as medicine, microvascular decompression or radio frequency. In this regard, transcranial direct current stimulation (tDCS) is another therapeutic method to reduce pain, which has been recently attracting the therapists’ attention. The positive effect of tDCS on TN was shown in many previous studies. However, the mechanism of the tDCS effect has remained unclear.

**Objective:**

This study aims to model the neuronal behavior of the main known regions of the brain participating in TN pathways to study the effect of transcranial direct current stimulation.

**Method:**

The proposed model consists of several blocks: (1) trigeminal nerve, (2) trigeminal ganglion, (3) PAG (periaqueductal gray in the brainstem), (4) thalamus, (5) motor cortex (M1) and (6) somatosensory cortex (S1). Each of these components is represented by a modified Hodgkin-Huxley (HH) model. The modification of the HH model was done based on some neurological facts of pain sodium channels. The input of the model involves any stimuli to the ‘trigeminal nerve,’ which cause the pain, and the output is the activity of the somatosensory cortex. An external current, which is considered as an electrical current, was applied to the motor cortex block of the model.

**Result:**

The results showed that by decreasing the conductivity of the slow sodium channels (pain channels) and applying tDCS over the M1, the activity of the somatosensory cortex would be reduced. This reduction can cause pain relief.

**Conclusion:**

The proposed model provided some possible suggestions about the relationship between the effects of tDCS and associated components in TN, and also the relationship between the pain measurement index, somatosensory cortex activity, and the strength of tDCS.

## Background

The TN (trigeminal neuralgia) is a rare facial pain disorder that leads to a sudden, short, and severe sense of pain in the face [1, 2]. It is one of the most severe neuropathic forms of pain [3]. This pain does not have a regular and normal behavior with a specific pattern. Therefore, the prediction of its occurrence is in a certain measure impossible. It may occur either spontaneously without doing any particular activity or by doing some routine tasks such as chewing, brushing teeth or even shaving, which can trigger the pain attack [1, 2].

Physiological factors (e.g., superior cerebellar artery compression) and plasticity of the nervous system have roles in TN to play [4]. Many diverse regions of the brain such as the thalamus, motor cortex (M1), brainstem, primary somatosensory cortex are included in the TN processing [5, 6], and connections and communications between these regions processing the TN, result in the TN network or the TN neuromatrix.

Carbamazepine, as one of the TN medicine treatments, can reduce the pain. However, the side effects of this medicine (e.g., drowsiness and confusion) usually result in discontinuation of its usage [1, 7]. Surgical interventions such as microvascular decompression or radiotherapies are other options that may be suggested to the patient with TN. Patients often do not tend to have surgery, because it has a high risk of face mutilation [1]. Transcranial direct current stimulation (tDCS) is another therapeutic method that was recently used in the field of pain and shows positive effect [1, 8]. This method is cheap and non-invasive. No serious side effect has been reported for this method.

The tDCS is a low direct current (usually 1 or 2 mA) which is applied to a specific region of the brain using two electrodes, which are placed on the superficial part of the brain. Motor cortex (M1) stimulation is more prevalent than in other regions of the brain. In this regard, M1 stimulation is utilized for pain relief, depression, addiction and so on [9, 10]. It was suggested that, by applying tDCS, pain perception is modulated by shifts of the resting membrane potential [1] and consequently results in the modification of the neuronal excitability at the stimulation site [1, 11]. Electrical stimulation (e.g., tDCS) of an appropriate area can play a role similar to that of the medial brain in reducing pain [4].

Despite the positive results of the effect of stimulation in pain relief, it is still unknown how tDCS can reduce the symptoms of TN. Modeling the pain pathway can provide a tool to understand some aspects of TN and to investigate the mechanism of tDCS effect. No computational model has been suggested for TN. However, there are some models of pain based on gate control theory [4] and artificial neural networks [12].

In the current study, we simplified a conceptual model of TN pathways that is proposed in the previous study [6]. Then we represented this conceptual model by a mathematical formula based on a modified version of the Hodgkin-Huxley (HH) equations. By using this model, the possible effects and mechanism of the influence of an external input such as tDCS were investigated. To evaluate the outcomes of our model, as we may not able to understand the meaning of S1 output potential or S1 activity outcome clearly, which is essential for investigating any modification in neuronal behavior in our model, we can change it to a more tangible and practical scale, such as visual analog scale (VAS) to comprehend the intensity of pain and the activity of S1. As a result, interpreting the output of our model, S1 activity is turned to VAS display, a method which is indeed efficient and practical for subjective measuring of pain, including TN. This self-evaluation scale ranges from 0 to 10 as visually described in centimeter units: 0 cm indicates no pain, and 10 cm means the worst pain possible. Participants will be asked to rate their pain during the previous 24 hours to get a baseline pain. This scale has been widely used in studies to evaluate pain as an outcome [13]. There are a few types of research which have done some experiments on TN patients by applying tDCS over M1 [1, 8, 14, 15].

In the next section, the details of the proposed conceptual and computational models are presented. The results of the simulation of the proposed model, considering the effect of tDCS, are described in the Results section. In the last part, the discussion and interpretation of the obtained results are provided as regards different computational and physiological aspects.

## Method

In this section, the stages of modeling have been described. At first, a simplified conceptual model of TN pathway has been introduced. This model, which has been explained in [6] in detail, consists of some important brain regions involved in TN. Then each part of the TN pathway has been modeled by a modified version of the HH model. At the last step, an external current stimulation has been applied over M1 to show the effect of external stimuli on TN.

### Trigeminal neuralgia pathway

**Figure 1:**
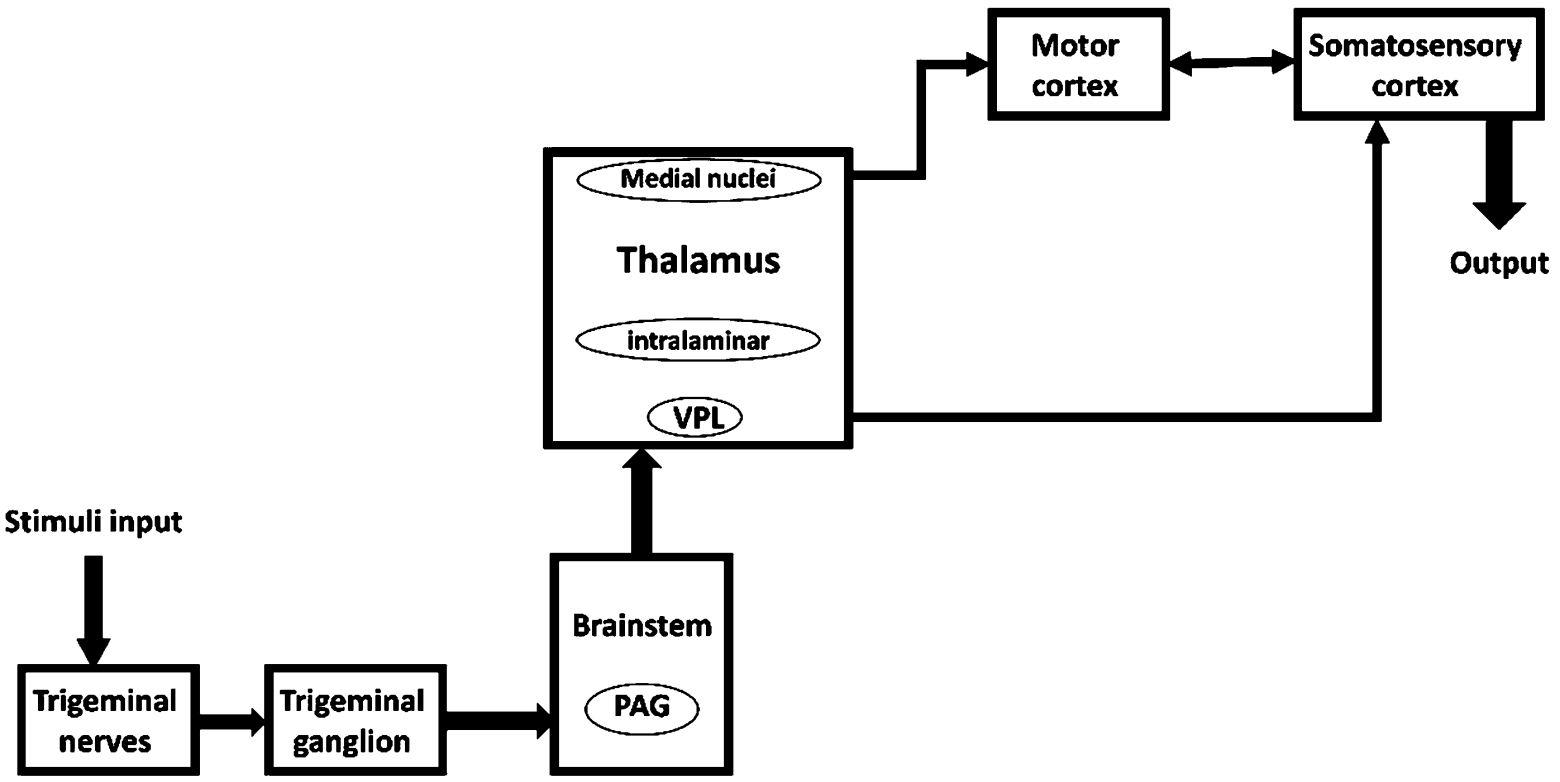
Concise TN pathway block diagram. PAG: periaqueductal gray, VPL: ventral posterolateral nucleus (reprinted from [6])

Many studies have investigated the brain regions involved in pain processing [1, 8, 16–18]. According to the results of these studies, there are a wide variety of brain areas that are involved in pain processing that can form a vast network with complex interactions. In our previous work [6], we have described this complicated network as a pain neuromatrix diagram. A simplified version of this neuromatrix is proposed that consists of the leading and substantial blocks of pain network in TN processing system from the initial noxious stimuli of TN to somatosensory cortex [11, 19–23]. The simplified pain neuromatrix model is shown in Fig. 1.

As shown in Fig. 1, this model includes the following blocks.

#### Trigeminal ganglion

Trigeminal neuralgia begins from the root of the nerve and trigeminal ganglion (TG) that is involved in the pain processing pathway. Somas of face neurons are in TG. The signals come from the face, and trigeminal afferents project using the TG, thereby they directly go to the brainstem and then project to the brain [16, 24].

#### Brainstem

After TG, the nociceptive signals reach to different parts of the brainstem [25, 26]. The brainstem consists of trigeminal nuclei [16, 18, 27–30], the para brachial (PB) nucleus, and PAG (periaqueductal gray). The brainstem projects signals to different nuclei of the thalamus [29, 31] especially the VPL (ventral posterolateral nucleus) and VPM (ventral posteromedial nucleus) regions [16, 19, 30].

#### Periaqueductal gray (PAG)

Periaqueductal gray is one of the substantial main parts of the pain-mediating process, which is in the middle part of the brainstem. It receives signals from thalamus [32], insula, and hypothalamus [31]. Periaqueductal gray involves the secretion of endogenous opioids, such as encephalin, for relieving pain [12, 19, 26, 28, 31–37].

#### Thalamus

The thalamus is one of the major structures that receives pain signals from diverse pain pathways [18, 19, 25, 26, 28–32, 34–43]. The thalamus processes the nociceptive information coming from the brainstem [29, 31] especially to the VPL and VPM regions [16, 19, 30] and projects them to different parts of the brain such as S2 (secondary somatosensory cortex) [37, 39, 40], primary somatosensory cortex (S1) [19, 30, 31, 37, 39, 40, 42] and PAG [32]. It has a reciprocal interaction with some parts of the M1 [35], especially the VL (ventral lateral nucleus) and anterior nuclei [36]. In this regard, it has been suggested that the thalamus may play a role in the inhibitory pain pathway by applying anodal tDCS over M1, which may result in a probable pain-relief effect [44].

#### Motor cortex

Although the primary motor cortex (M1) is not considered regularly as part of the pain neuromatrix, it plays a crucial role in modulating the pain in different chronic pain syndromes [25, 28, 35–37, 41, 45]. It has some reciprocal connections with S1 [28, 37, 45]. It receives direct information from the ACC (anterior cingulate cortex) [41] and sends it to the prefrontal cortex [25], brainstem [25, 26] and thalamus [25, 26], and especially VPL [28]. Many studies signify the importance and effects of the tDCS over M1 and put emphasis on the role of motor cortex stimulation in pain intensity reduction or increase in the pain threshold [1, 8, 14, 25, 26, 46–49]. Although the mechanism of the effect of the M1 anodal tDCS has remained somewhat unclear, such pain-relief effects may exist because of sub-cortical and thalamocortical connections [44].

#### Somatosensory cortex

The primary somatosensory cortex is also one of the main cortical regions in the pain or TN neuromatrix [5, 16, 19, 28–31, 35–37, 39–42, 45, 50, 51]. The primary somatosensory cortex has some mutual interaction with M1 [28, 37, 45] and S2 [37]. The primary somatosensory cortex receives nociceptive information from S2 [41], and the thalamus [19, 30, 31, 37, 39, 40, 42].

In the above paragraphs, a brief review of the simplified pain neuromatrix model was provided. More details can be found in [6]. In the next section, this model has been formulated by mathematical equations.

### Mathematical modeling of the simplified pain neuromatrix

The Hodgkin–Huxley model gives the ability to investigate the chemical reactions and activity changes of neuronal response. The equations that describe the HH model can be found in textbooks.

It has been shown that some ion channels, such as the Na_v_1.8 slow sodium channels, play a role in pain pathway and pain intensity modification. In this regard, their synthesis and activity may also cause different neuronal potential and behavior [52]. The HH model has the capability to model and describe the effect of diverse factors influencing the ion channels. Moreover, the equations presented for the HH model can take into account the activity variation of neuronal behaviors. Importantly, use-dependent sodium channel inhibitors are clinically effective in the treatment of many types of chronic pain [53]. Hyperalgesia is removed by factors decreasing impulse activity of Na_v_1.8 channels. That is why these factors are believed to be of use in highly selective pain-killing medicine [52]. Considering the physiological role of the activation gating structure of the slow sodium channels Na_v_1.8 in impulse coding of nociceptive information [54], and observing that the modification of specified slow sodium channels in the membrane of nociceptive neurons is the basis of the pain perception [52], it seems that the HH model is able to be a proper candidate for modeling the pain modulation process. However, it needs some modifications for using in our pain processing study. A voltage-gated slow Na^+^ current needs to be added into the HH equations. In other words, despite HH original model being useful for modeling the behavior of neurons, it is a general model and should be specialized for our use in pain-related neurons and simulating their behavior. Considering one more ion channel will definitely result in a more realistic simulation, since we have separated the current and gating variables related to it in our model. Besides, it is necessary to understand what parameters cause the possibility of the nociceptive neuron to *affect* generating or preventing a painful signal. As a result, the extra current for pain intensity plus its corresponding activity fluctuation needs to be considered in the HH model. In fact, the added current is the Na_v_1.8 slow sodium channel current specified for pain and pain modulation processing [52]. Therefore, the modifications have been applied by adding two more equations to the main HH equations (Eqs. (5) and (6)). The modified version of the HH (MHH) model is described by Eqs. (1)–(16):

1 $$\begin{aligned}& C_{m}\frac{dE}{dt} = I - g_{Naf}m^{3}h(E - E_{Na}) - g_{k}n^{4}(E - E _{K}) - g_{L}(E - E_{L}) - g_{NaS}m_{S}^{3}h_{S}(E - E_{Na}), \end{aligned}$$

2 $$\begin{aligned}& \frac{dm}{dt} = \alpha _{m}(E) (1 - m) - \beta _{m}(E)m , \end{aligned}$$

3 $$\begin{aligned}& \frac{dh}{dt} = \alpha _{h}(E) (1 - h) - \beta _{h}(E)h, \end{aligned}$$

4 $$\begin{aligned}& \frac{dn}{dt} = \alpha _{n}(E) (1 - n) - \beta _{n}(E)n, \end{aligned}$$

5 $$\begin{aligned}& \frac{dm_{s}}{dt} = \alpha _{m_{s}}(E) (1 - m_{s}) - \beta _{m_{s}}(E)m _{s}, \end{aligned}$$

6 $$\begin{aligned}& \frac{dh_{s}}{dt} = \alpha _{h_{s}}(E) (1 - h_{s}) - \beta _{h_{s}}(E)h _{s}, \end{aligned}$$

where

7 $$\begin{aligned}& \alpha _{m}(E) = \frac{0.115(1 + e^{\frac{E + 70}{10}})}{e^{\frac{E + 40}{42}} + 1}, \end{aligned}$$

8 $$\begin{aligned}& \beta _{m}(E) = 0.015\bigl(1 + e^{\frac{E + 25}{8}}\bigr), \end{aligned}$$

9 $$\begin{aligned}& \alpha _{h}(E) = 0.012\bigl(1 + e^{\frac{ - (E + 43)}{10}}\bigr), \end{aligned}$$

10 $$\begin{aligned}& \beta _{h}(E) = \frac{1.32}{1 + 0.2e^{\frac{(E + 10)}{7}}}, \end{aligned}$$

11 $$\begin{aligned}& \alpha _{n}(E) = \frac{0.006(E + 45)}{1 - e^{\frac{E + 45}{12}}}, \end{aligned}$$

12 $$\begin{aligned}& \beta _{n}(E) = 0.13\bigl(e^{\frac{ - (E + 45)}{30}}\bigr), \end{aligned}$$

13 $$\begin{aligned}& \alpha _{mS}(E) = \bigl(e^{0.0769(E) - 0.553}\bigr), \end{aligned}$$

14 $$\begin{aligned}& \beta _{mS}(E) = \bigl(e^{ - 0.00029(E) - 2.523}\bigr), \end{aligned}$$

15 $$\begin{aligned}& \alpha _{hS}(E) = 0.0015\bigl(e^{\frac{ - (E + 40)}{30}}\bigr), \end{aligned}$$

16 $$\begin{aligned}& \beta _{hS}(E) = \biggl(\frac{0.1}{1 + 0.2e^{\frac{ - (E + 10)}{7}}}\biggr). \end{aligned}$$

The parameters and variables used in Eqs. (1)–(16) have been introduced in Table 1. These equations are voltage-dependent expressions which are extracted from previous studies [52]. HH model parameters are obtained by space-clamped experiments.

**Table 1:** Variables and parameters definitions

Variable or paremeter name	Definition
*E*	Membrane potential
*m*	Activation of fast sodium channels
*h*	Inactivation of fast sodium channels
$m_{{s}}$	Activation of slow sodium channels
$h_{{s}}$	Inactivation of slow sodium channels
*n*	Activation of potassium channels
$C_{{m}}$	Equivalence membrane capacitance
$g_{{Naf}}$	Fast sodium channels conductance
$g_{{k}}$	Potassium channels conductance
$g_{{l}}$	Leak channels conductance
$g_{ {NaS} }$	Slow sodium channels conductance
$E_{{Na}}$	Nernst potentials of fast sodium ions
$E_{{k}}$	Nernst potentials of potassium ions
$E_{{l}}$	Nernst potentials of leakage ions
$\alpha _{{m}}$ and $\beta _{{m}}$	Transition rates between open and closed states of the activation of sodium channels
$\alpha _{{h}}$ and $\beta _{{h}}$	Transition rates between open and closed states of the inactivation of sodium channels
$\alpha _{{n}}$ and $\beta _{{n}}$	Transition rates between open and closed states of the potassium channels
$\alpha _{{mS}}$ and $\beta _{{mS}}$	Transition rates between open and closed states of the activation of Slow sodium channels
$\alpha _{hS}$ and $\beta _{hS}$	Transition rates between open and closed states of the inactivation of slow sodium channels

**Figure 2:**
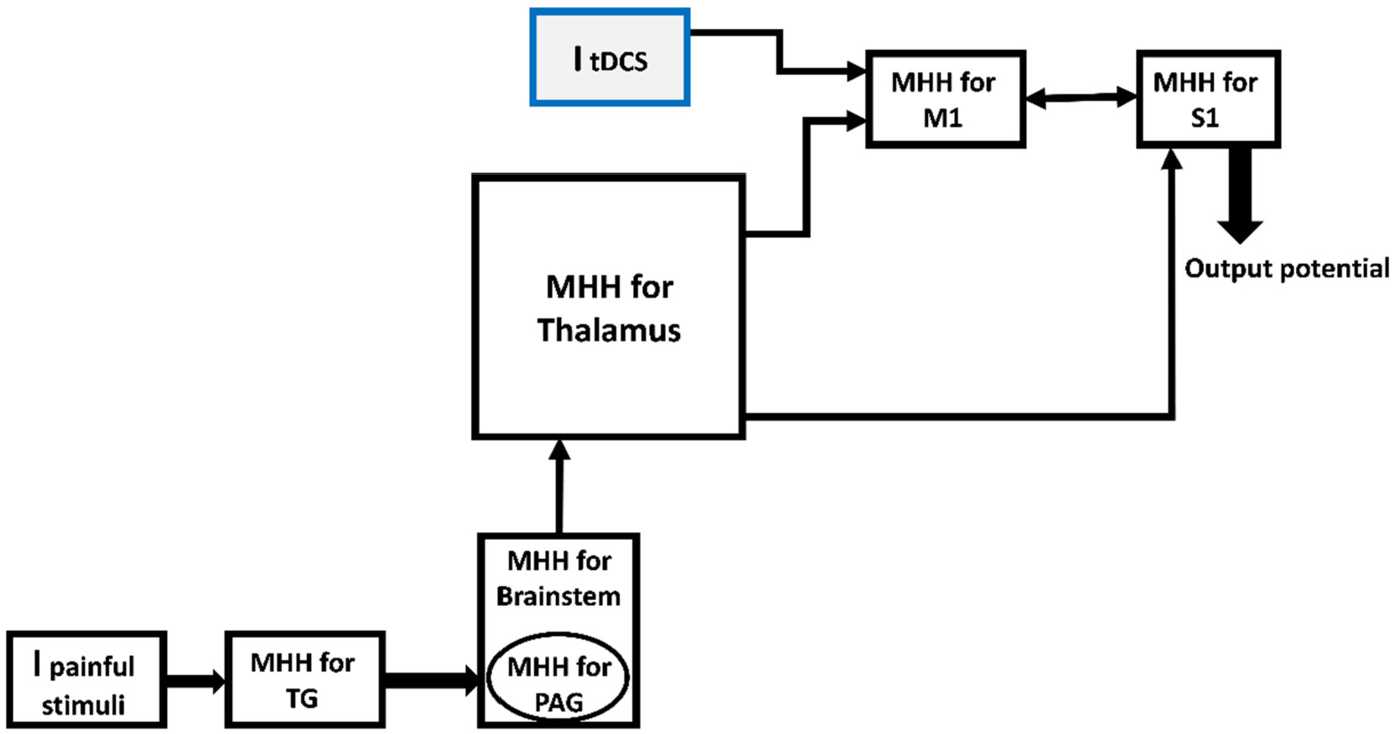
Embedding MHH for each block and inserting ItDCS to M1. MHH: modified Hodgkin Huxley, TG: trigeminal ganglion. PAG: periaqueductal, M1: motor cortex, S1: somatosensory cortex

As shown in Fig. 2, to formulate the simplified pain neuromatrix model (Fig. 1), each block was modeled by MHH equations. The input current (*I*) is the noxious stimuli. So, this input was applied to the region where the pain started (i.e., TG). The characteristics of TN, such as high intensity of pain, shock-likeness and discontinuity of occurrence, have been represented by the features of the input (*I*).

Each block of the model is differentiated from the other ones by considering its specific characteristics such as the membrane capacitances, initial membrane potentials, and conductivities of channels that are proportional to initial potentials of the block. The conductance of each block can be varied from one block to another; however, without losing the whole issue, we considered the same conductance for each block for simplicity, which can be calculated by separate studies for each part of the brain in future research and be as precise as validated experiments. In other words, the same conductance for each block (except the trigeminal ganglion) is considered regarding the Gabriel paper [55] for electrical conductivity of body tissues, and the values in the model and because of simplicity, as mentioned. The MHH applied for each block is the following.

At first, the initial values for reversal potentials related to each channel, the maximum of the conductance of them, the initial potential for the first block and the gating variables were defined. Then, at the beginning of each block, there were different Alphas and Betas related to each block was calculated. Then these Alphas and Betas were applied to the related gating variables such as m, h, n, mS, and hS. After calculating the gating variables, the conductivity of each channel and then the specified current related to each channel was calculated. At last, the output potential of the block was obtained by considering the (I)-form of MHH (Eq. (11)) and the input stimuli (or current for other blocks except the first one). So the input current of the next block was calculated from the output potential of the previous block multiplied by the conductance of that block, which results in the current (I)-form of the next block and these procedures continue till obtaining the output potential (or activity) of S1 block. The values have been shown in Table 2.

**Table 2:** Specific values used for simulation

Variable or parameter name	Definition
$C_{{m}}$	5 pF [54]
$g_{Naf}$	25 nS [54]
$g_{{k}}$	20 nS [52, 54]
$g_{{l}}$	5 nS [52]
$g_{ {NaS} }$	100 nS [52]
$E_{Na}$	60 mV [54]
$E_{{k}}$	−75 mV [54]
$E_{{l}}$	−55 mV [54]
$\varepsilon _{r\text{ (TG)}}$	2.01∗10^7^ [55]
$\varepsilon _{r\text{ (all blocks except TG)}}$	4.07∗10^7^ [55]
$\varepsilon _{0}$	8.854∗10^−12^
$C_{m\text{ (TG)}}$	5 pF [54]
$C_{m\text{ (all blocks except TG)}}$	10.1245 pF (using Eq. (17))
*G*	$\frac{1}{2.2\mbox{ ($\varOmega$ m)}} = 0.45(\frac{S}{m})$ [56]

The output *potential* (*E*) of each block is the input of the next block. Therefore, it is required to reform the output potential (*E*) to input current (*I*) of the next block. The mentioned conductance (*G*) is used to transform the output potential (*E*) form of the previous block to the input current form (*I*) of the next block (i.e., $I = G * V$).

The *capacitance* of the blocks is calculated from Eq. (17),

17 $$ C = \varepsilon _{0}\varepsilon _{r}\frac{A}{d}. $$

Here $\varepsilon _{0} = 8.854*10^{ - 12}$ and $\varepsilon _{r}$ are the absolute permittivity and relative permittivity of the selected region (i.e., each block) of the brain, respectively. *A* is the area of the membrane cross-section, *d* is a separation between intra- and extracellular.

The proposed model has been simulated considering parameters amounts that have been reported in the next part.

Matlab R2013b with SCR:001622 RRID number was used as a software tool.

## Results

In the equations which were described in the previous section, the values of the parameters have been selected as indicated in Table 2. The numbers are based on some formula and the amounts reported in previous studies, which have been mentioned next to each value.

**Figure 3:**
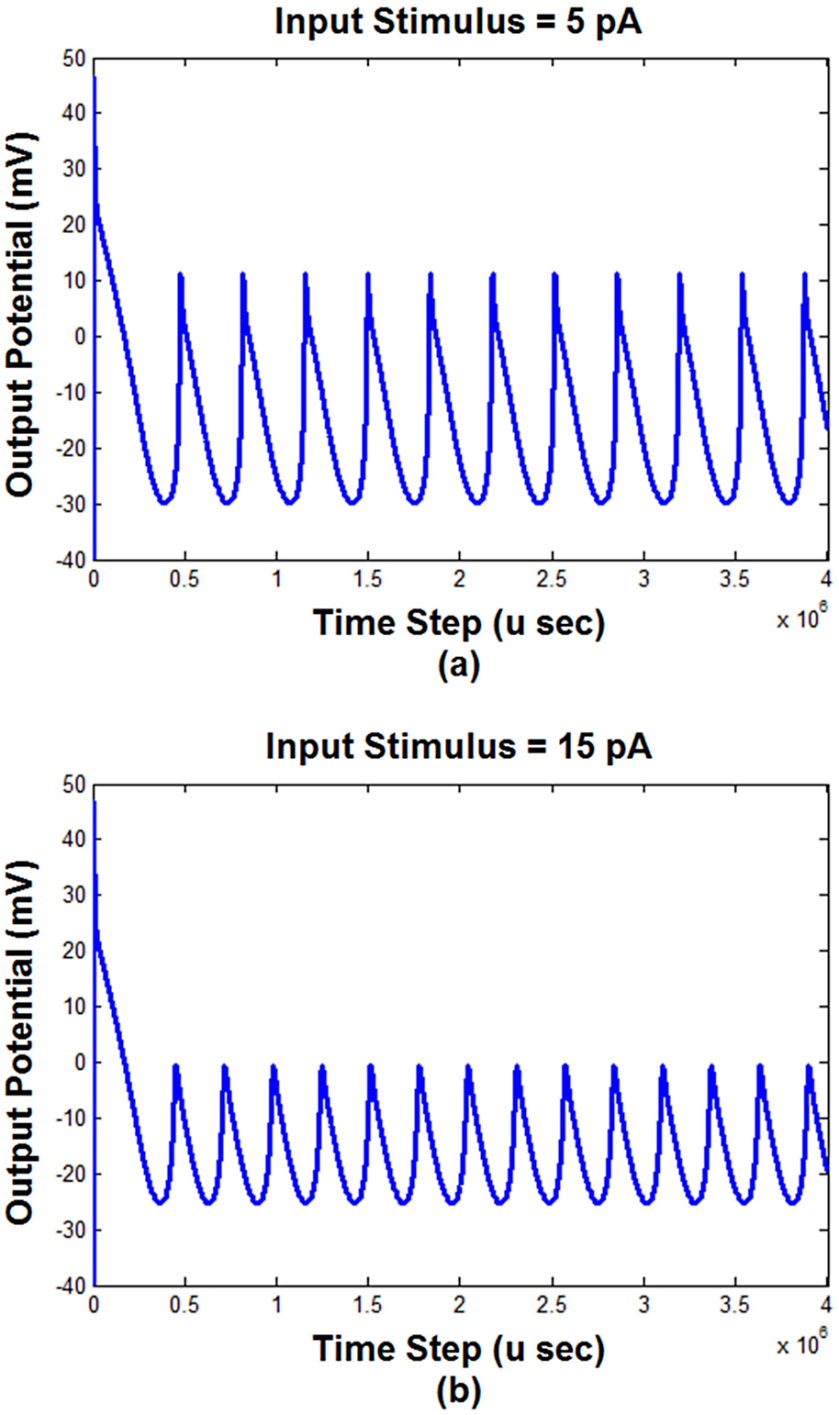

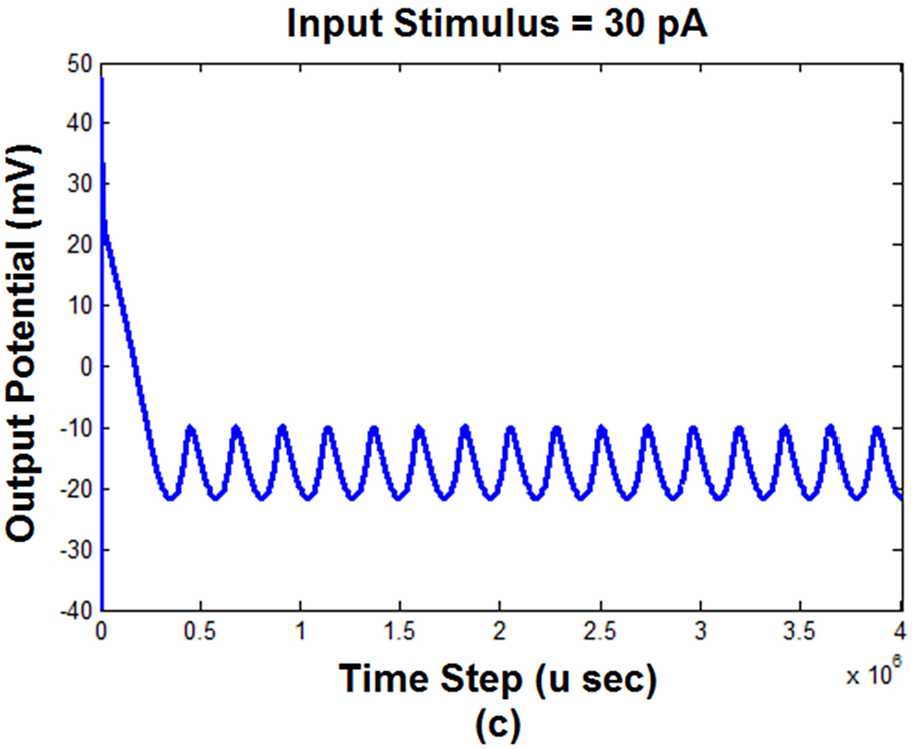
The response of the first block of the model (TG) to the input stimulus with different amplitudes ((**a**) $\mbox{input stimulus} = 5\mbox{ pA}$; (**b**) $\mbox{input stimulus} = 15\mbox{ pA}$; (**c**) $\mbox{input stimulus} = 30\mbox{ pA}$)

As shown in Fig. 2, the input stimulus is applied to the TG block. As a result, the simulation indicates that the amplitude of the input affects the output behavior of this block that is shown in Fig. 3.

Figure 3 demonstrates that applying a different amount of input stimulus changes the behavior of the output of the first block. According to Fig. 3, the peak to peak value of the output decreased by increasing the input stimulus. Regardless of the transient part of the output, the increment of the input stimulus led to the increment of the minimum value of the output and decrement of the maximum value. Increasing the input amplitude had no considerable effect on the transient part. However, it seems that the upward slope of the output changes declined by the increment of the input stimulus. When the input amplitude is 5 pA, Fig. 3(a) shows that the output has 11 peaks (cycles) during the time of the stimulation (i.e., length of the horizontal axis). According to Fig. 3(b), increasing the input to 15 pA, the number of peaks increased to 14. The more increment of the input to 30 pA led to the changes in the peak’s number from 14 to 16 (Fig. 3(c)). Therefore, it can be concluded that increasing the input amplitude (i.e., the strength of the pain) affects the activity and the amplitude of the TG output.

**Figure 4:**
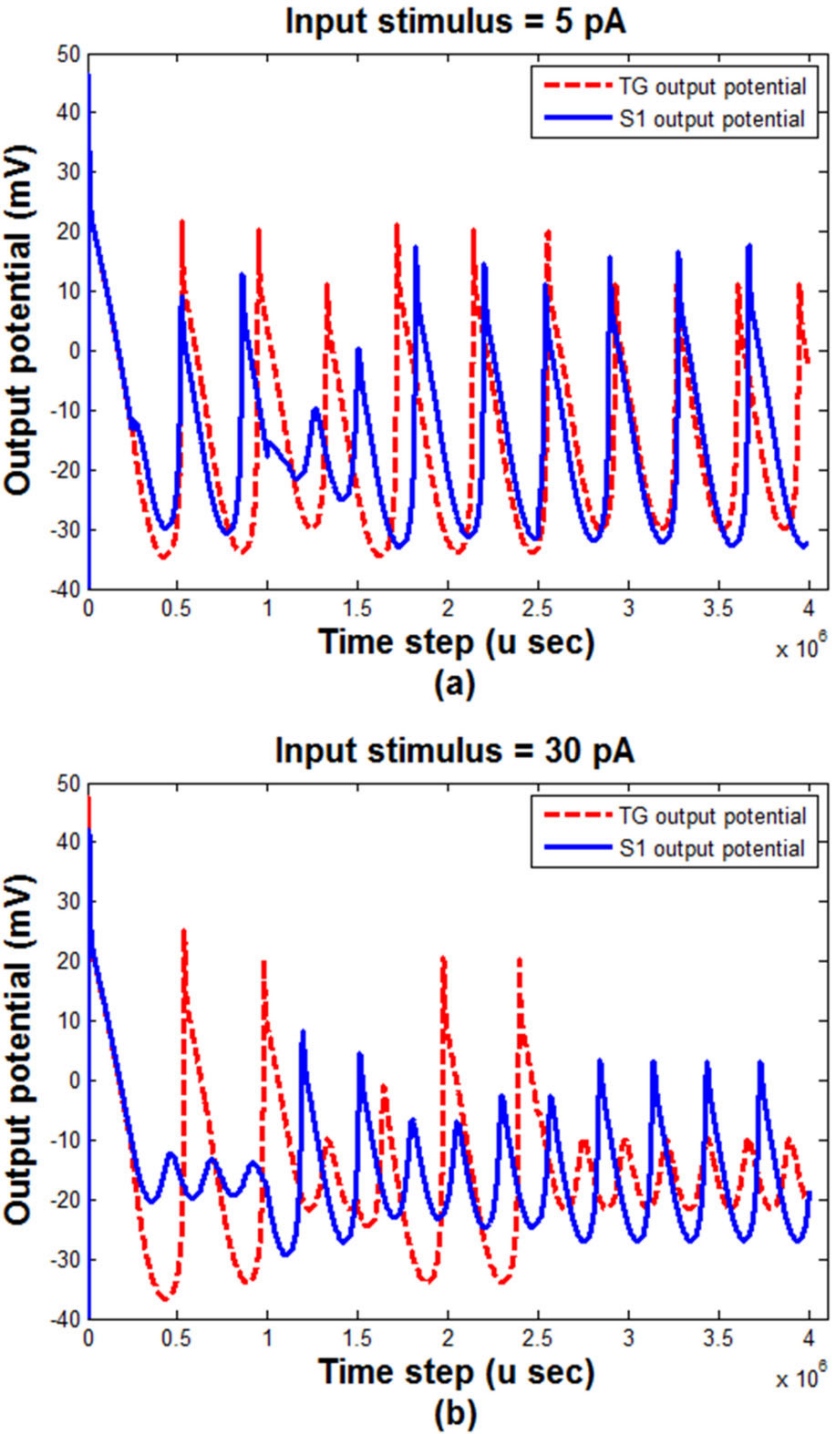
The output of TG and S1 blocks with different input stimulus maximum amplitudes. (**a**) $\mbox{Maximum amplitude} = 5\mbox{ pA}$, (**b**) $\mbox{maximum amplitude} = 30\mbox{ pA}$. TG: trigeminal ganglion, S1: somatosensory cortex

According to the outcomes of previous studies, ordinary pain can be simulated by a constant input current [52, 54]. Figure 3 showed the behavior of the TG block to a common pain (i.e., constant current). However, the pain of TN does not have a regular shape. It seems that its pattern changes randomly [1, 2, 57]. Therefore, to simulate such a pain, we have considered an input current that randomly fluctuated between zero and a positive or negative value. Figure 4 shows the output of TG and S1 (i.e., the output of the model) blocks to this random input with two maximum values.

As shown in Fig. 4, considering the TN pain the output of the TG block is not as regular as the common pain (Fig. 3). It can be seen that increasing the range of the changes of the input (i.e., TN pain) led to the decrement of the sum squares of the outputs of both TG and S1 blocks. It has also been observed that the phase difference between the output of TG and S1 increases as the range of the input changes is increased.

**Figure 5:**
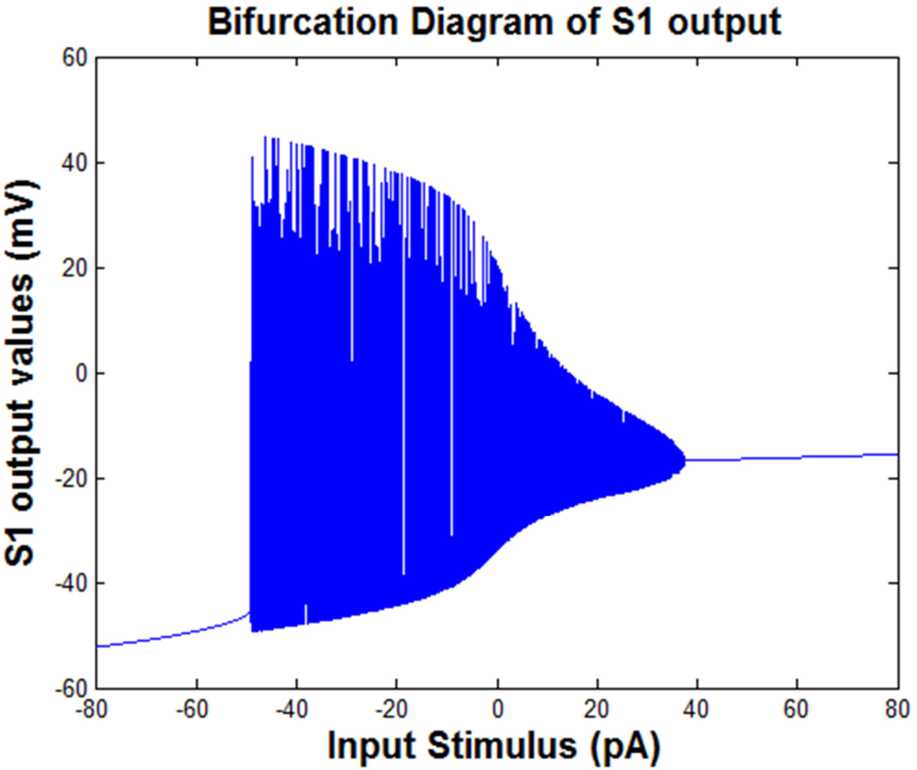
The bifurcation diagram of the values of the S1 output. The range of the input changes is the control parameter ($g_{NaS} = 100\mbox{ nS}$)

Figure 5 shows the bifurcation diagram of the extreme values of the S1 output (i.e., the model’s output) considering the range of the input changes as the control parameter. The conductivity of the slow sodium channel was $g_{ {NaS} }=100\mbox{ nS}$.

According to Fig. 5, increasing the range of the input changes to higher than about 40 pA and decreasing this value to lower than about −50 pA led to a regular (i.e., harmonic) output. Between these two values (i.e., −50 pA and 40 pA), the output behavior is bursting and increasing the range of the changes of the input stimulus leading to the decrement of the range of the changes of the S1 output. Acceding to the middle part of the diagram (i.e., where the input is between −50 pA to 40 pA), it can be seen that the minimum value of the output decreases and the maximum value increases by the increment of the range of input changes. Different values have been considered for input (stimuli) current from $I0= -80$ to 80 in order to observe the behavior of S1 behavior with a fixed value of conductivity of slow sodium channels ($g_{ {NaS} }$) for Fig. 5. The size of the output potential of S1 was calculated in rows and columns. Then, for each *I*0 value mentioned and the related column of the output potential of S1 with the whole row of that column, the figure was plotted. The entire *I*0 value in correspondence to the S1 output potential was created by holding command on each plotting.

**Figure 6:**
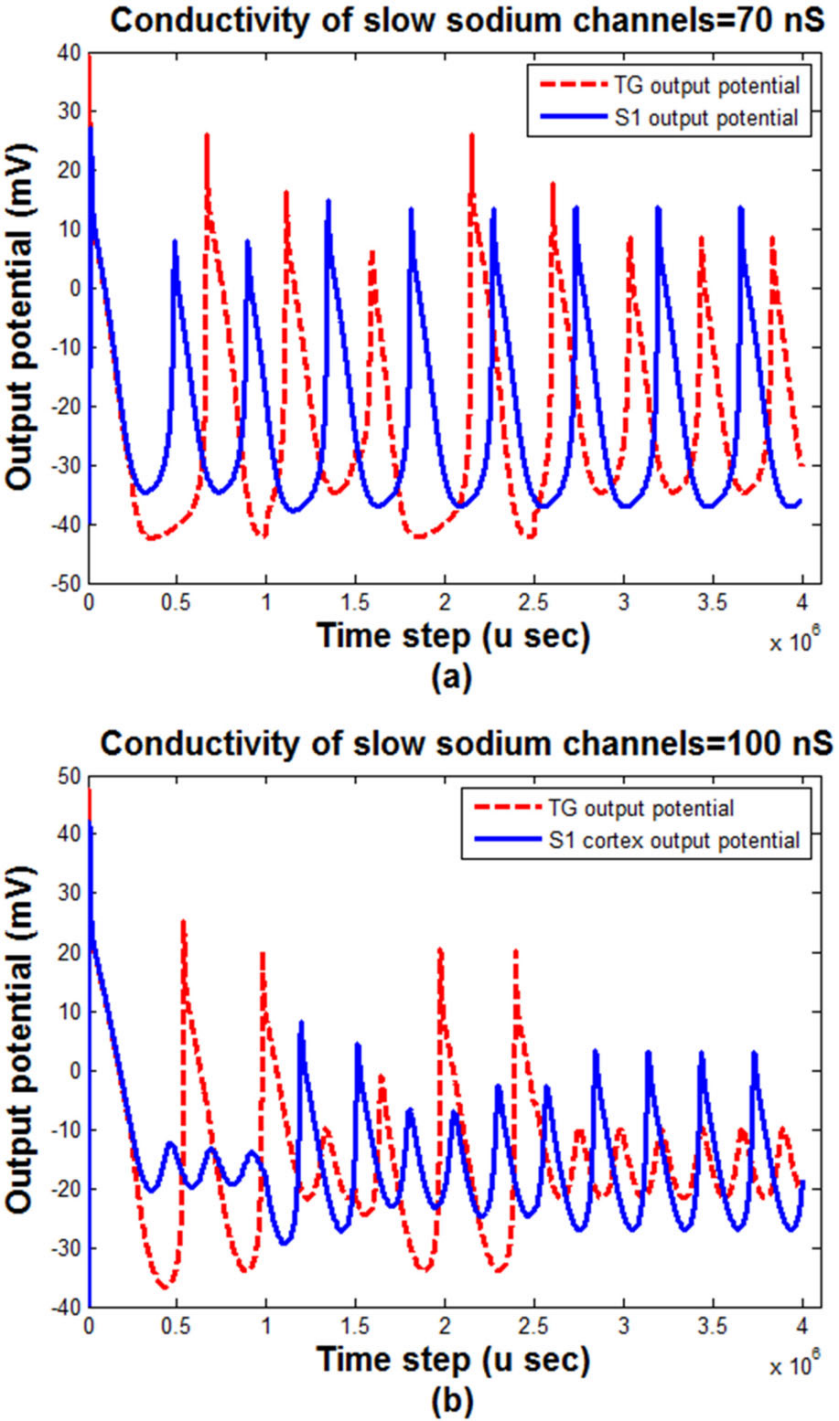
The effect of the conductivity of slow sodium channels on the output pattern of the TG and S1 blocks. (**a**) $\mbox{Conductivity} = 70\mbox{ nS}$, (**b**) $\mbox{conductivity} = 100\mbox{ nS}$; $\mbox{input stimulus range} = (0,30\mbox{ pA})$

In addition to the input stimulus, the conductivity of channels can affect the pattern of the output of the model. Figure 6 shows the effect of the conductivity of slow sodium channels on the output of TG and S1 blocks.

According to Fig. 6, the sum squares of the outputs of both TG and S1 blocks is decreased by the increasing of slow sodium channels conductivities named $g_{ {NaS} }$. The phase difference between the outputs of TG and S1 blocks is more obvious in the lower value of the slow sodium channels conductivities than the higher one.

**Figure 7:**
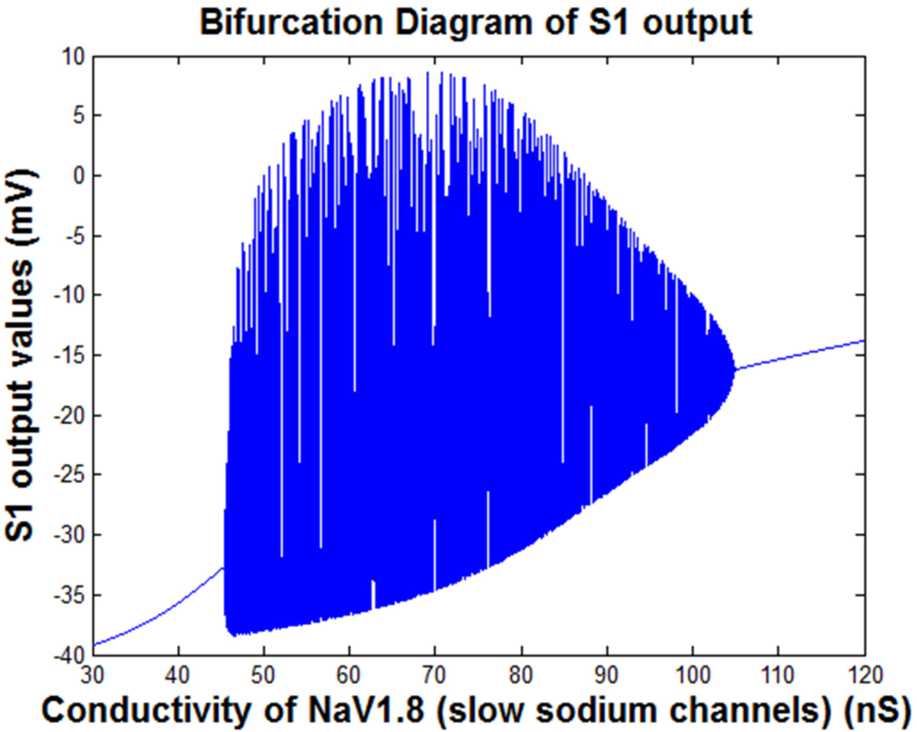
The bifurcation diagram of the extreme values of the S1 output. The slow sodium channels conductivity ($g_{ {NaS} }$) is the control parameter ($\mbox{input stimulus}=30\mbox{ pA}$)

The bifurcation diagram of the values of the S1 output considering the slow sodium channels conductivity as the control parameter is shown in Fig. 7. The input stimulus is considered 30 pA.

Figure 7 was created by considering different values for the conductivity of slow sodium channels $g_{ {NaS} }$ from $g_{ {NaS} } =30$ to 120 in order to observe the behavior of S1 behavior with a fixed value of input stimuli for Fig. 7. The size of the output potential of S1 was calculated into rows and columns. Then for each $g_{ {NaS} }$ the mentioned value and the related column of the output potential of S1 with the whole row of that column, the figure was plotted. The entire $g_{ {NaS} }$ value in correspondence to the S1 output potential was created by holding command on each plotting.

As shown in Fig. 7, the bifurcation diagram of the S1 output has three different parts. The first and last parts exhibit a harmonic behavior (with one frequency component) of the output. The amplitude of this harmonic behavior increases by increasing the $g_{ {NaS} }$. In the middle part of the diagram, the output consists of a variety of frequency component (i.e., bursting behavior) and the minimum value of the output decreases by the increment of the range of input changes. However, the maximum amount of the output has an inverse-U shape. It increases and then decreases by increment of the input range.

### Nonlinear dynamic analysis of the model

Different types of bifurcations have been considered previously in the *bifurcation of HH equations* study by Guckenheimer [58] in which a qualitative depiction of the different regimes of the bifurcation diagrams for HH in the two-dimensional I-V_K_ parameter plane, limit cycles, diagrams and phase portraits on the two-dimensional invariant manifold for HH on the I-V_K_ plane have been obtained. In another study, *chaos in the HH model* [59], the phase space of the HH model has been described which results in the existence of a degree of unpredictability about how the system will respond to stimulation. It has been concluded that the results established the subtlety of the concept of threshold which says: “*the excitability of a neural membrane to fire an action potential may be more complex than a smooth hypersurface that divides subthreshold and supra-threshold membrane potentials*.”

**Figure 8:**
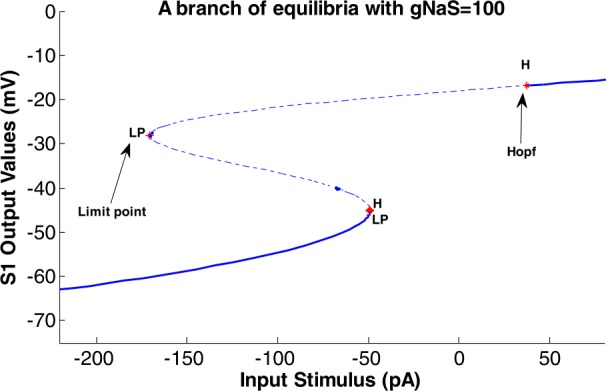
A branch of equilibria in the (*I*0-*S1 output*)-plane displaying Hopf bifurcations with $g_{ {NaS} }=100$

**Figure 9:**
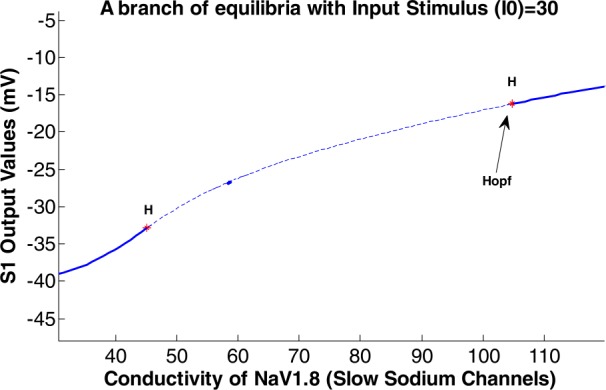
A branch of equilibria in the ($g_{ {NaS} }$-*S1 output*)-plane displaying Hopf bifurcations with input stimulus (*I*0) =30

The nonlinear dynamics of the mentioned modified HH model resulting in its different behaviors are shown in Figs. 5 and 7. Using the MatCont, a standard bifurcation software, the type of bifurcation that system encounters is shown in Fig. 8 for varying intensity of the input stimulus (I0), and Fig. 9 for varying conductivity of the slow sodium channels ($g_{ {NaS} }$).

Figure 8 has been obtained by activating the parameter *I*0 in the Equilibrium part of MatCont. The stability changes upon the variation of *I*0 and thus we could also see how the eigenvalues of the Jacobian at the equilibrium develop.

We have the following information from the indicated points in Fig. 8.

When $I0 = 37.416140$ is reached a pair of complex conjugate eigenvalues and a Hopf bifurcation occur. By considering the negative $\mbox{first Lyapunov coefficient} = -4.294451\mbox{e}{-}04$, the resulting Hopf is stable.

$$\begin{gathered} \text{label} =H,\\ x= \begin{pmatrix} -16.826666& 0.993461& 0.009702& 0.786201& 0.661761& 0.260367& 37.416140 \end{pmatrix}, \\ \text{First Lyapunov coefficient} = -4.294451\mbox{e} {-}04. \end{gathered} $$

At $I0 = -170.355702$ the message ‘Limit point’ appears which shows one eigenvalue has a positive real part. The quadratic coefficient (normal form coefficient) is given as ‘*a*’.

$$\begin{gathered} \text{label} =LP,\\ x= \begin{pmatrix} -28.153602& 0.992439& 0.011037& 0.643778& 0.449380& 0.552195& -170.355702 \end{pmatrix}, \\ a=-3.838174\mbox{e} {-}04. \end{gathered} $$

When $I0 = -49.126377$ is reached two pairs of complex conjugate eigenvalues and a Hopf bifurcation occur. The MatCont named this point ‘Neutral saddle’, which has two real eigenvalues with opposite sign.

$$\begin{gathered} \text{label} =H,\\ x= \begin{pmatrix} -45.015326& 0.980167& 0.019813& 0.356172& 0.181721& 0.947642& -49.126377 \end{pmatrix}, \\ \text{Neutral saddle}. \end{gathered} $$

At $I0 = -49.120424$ another ‘Limit point’ is found. ‘*a*,’ in both Limit points, is involved in the non-degeneracy condition and has been computed automatically.

$$\begin{gathered} \text{label} =LP,\\ x= \begin{pmatrix} -45.109623& 0.980035& 0.019914& 0.354551& 0.180641& 0.948439& -49.120424 \end{pmatrix}, \\ a=2.891546\mbox{e} {-}02. \end{gathered} $$

The two Limit Point bifurcations are shown, and *x* indicates the six variables *E*, *m*, *h*, *n*, *ms*, *hs* and the value of the active parameter *I*0 at the bifurcation point.

In Figs. 8 and 9, the limit cycles that were born from the first and second Hopf bifurcation can be unstable (dotted line) and stable (continuous line) as the first Lyapunov coefficient is positive and negative, respectively.

When $g_{ {NaS} } = 45.162360$ is reached, two pairs of complex conjugate eigenvalues and a Hopf bifurcation occur. The negative $\mbox{first Lyapunov coefficient} = -9.946008\mbox{e}{-}05$ shows the Hopf point is stable.

$$\begin{gathered} \mbox{label} =H,\\ x= \begin{pmatrix} -32.861315& 0.990762& 0.012243& 0.568873& 0.362030& 0.710132& 45.162360 \end{pmatrix}, \\ \mbox{First Lyapunov coefficient} = -9.946008\mbox{e} {-}05. \end{gathered} $$

At $g _{{NaS}} = 104.772243$ a pair of complex conjugate eigenvalues and another Hopf bifurcation occur. The negative $\mbox{first Lyapunov coefficient} = -4.731031\mbox{e}{-}04$ demonstrates a stable Hopf point.

$$\begin{gathered} \mbox{label} =H,\\ x= \begin{pmatrix} -16.229848& 0.993428& 0.009668& 0.792147& 0.671994& 0.251113& 104.772243 \end{pmatrix}, \\ \mbox{First Lyapunov coefficient} = -4.731031\mbox{e} {-}04. \end{gathered} $$

**Figure 10:**
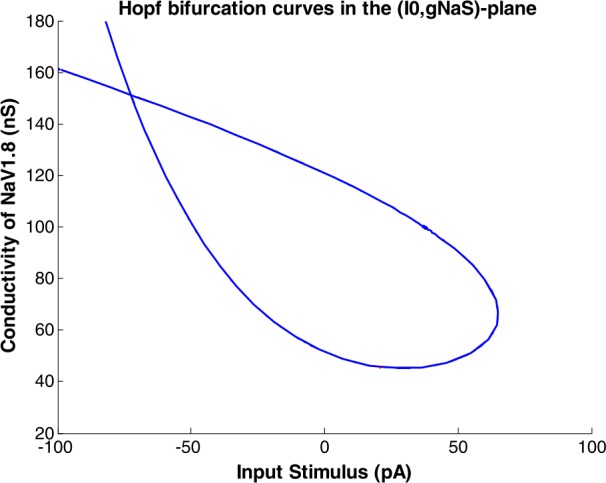
Hopf bifurcation curves in the ($I0 -g_{ {NaS} }$)-plane

By selecting one of the Hopf cases, shown in Fig. 8, we found in the equilibrium continuation the initial point and activating *I*0 and $g_{ {NaS} }$ as active parameters, and changing the curve type to Hopf from ‘Type’ menu, Fig. 10 has been obtained.

By drawing the $I0-g_{ {NaS} }$ plane, inside the obtained oval-shaped figure, the system behaves differently in comparison to the outside of it. In other words, while we choose an *I*0 and a $g_{ {NaS} }$ from the inside of the oval-shaped figure, the output values of the S1 shows oscillation and a periodic behavior will be presented, and by increasing the input stimulus, the activity of the output of S1 will be increased as well. On the other hand, while we choose a point outside the oval-shaped figure, no periodic behavior can be seen and the output of S1 will show constant values.

**Figure 11:**
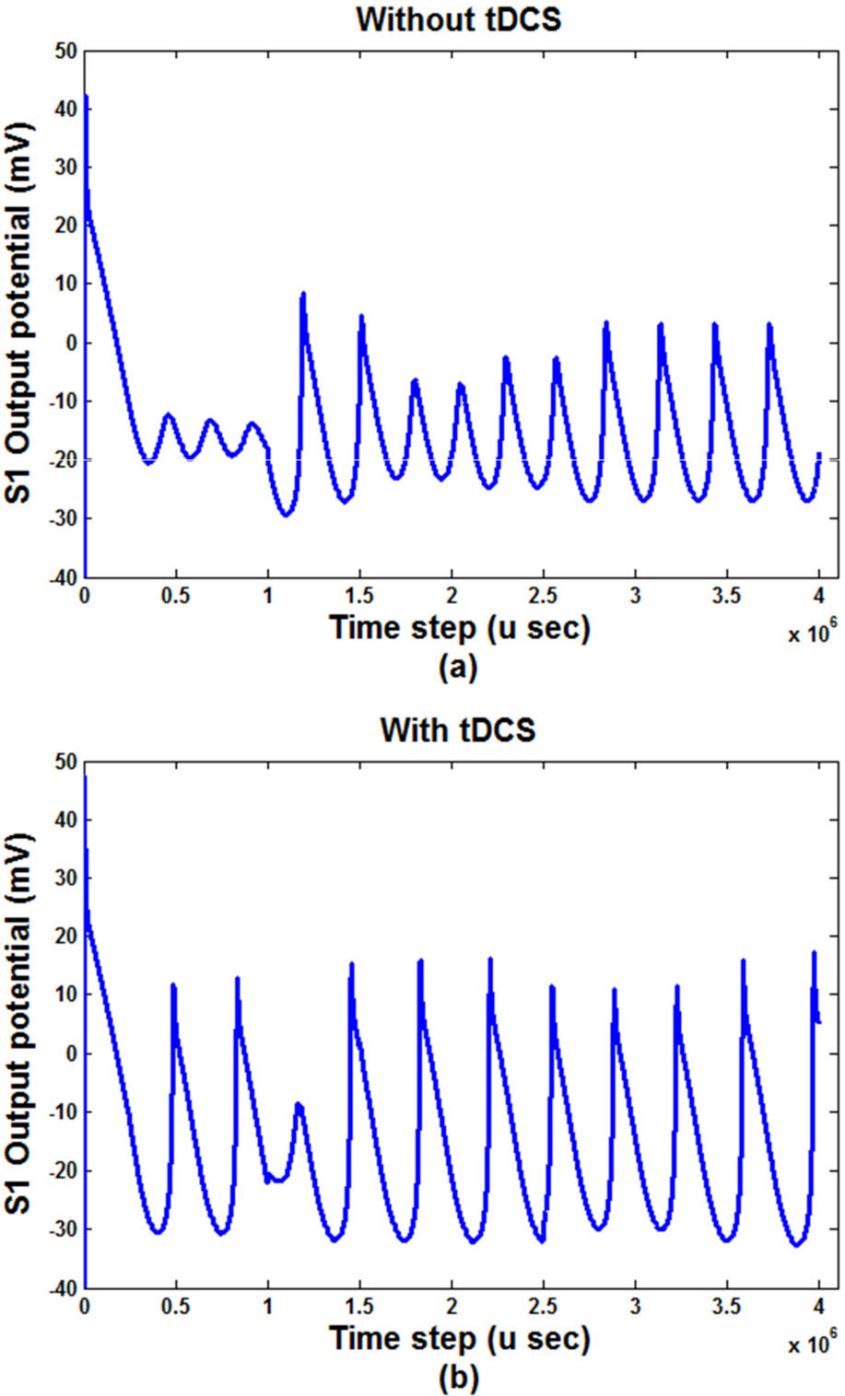
Output behavior of S1 (**a**) without ItDCS, (**b**) with ItDCS

The previous simulations were for a model without considering an external current stimulus to M1. By adding another current as an external input current, which can be regarded as a tDCS current ($I_{tDCS}$) to M1 block (see Fig. 2), the results of simulations can show the effect of $I_{tDCS}$ on the output behaviors. Figure 11 shows the outputs behavior (i.e., the output of TG and S1 blocks) with and without considering $I_{tDCS}$. In this figure, the input stimulus is 30 pA, and the conductivity of pain channels is $g_{ {NaS} }=100$.

As shown in Fig. 11, the sum squares of the S1 output increased by adding $I_{tDCS}$.

**Figure 12:**
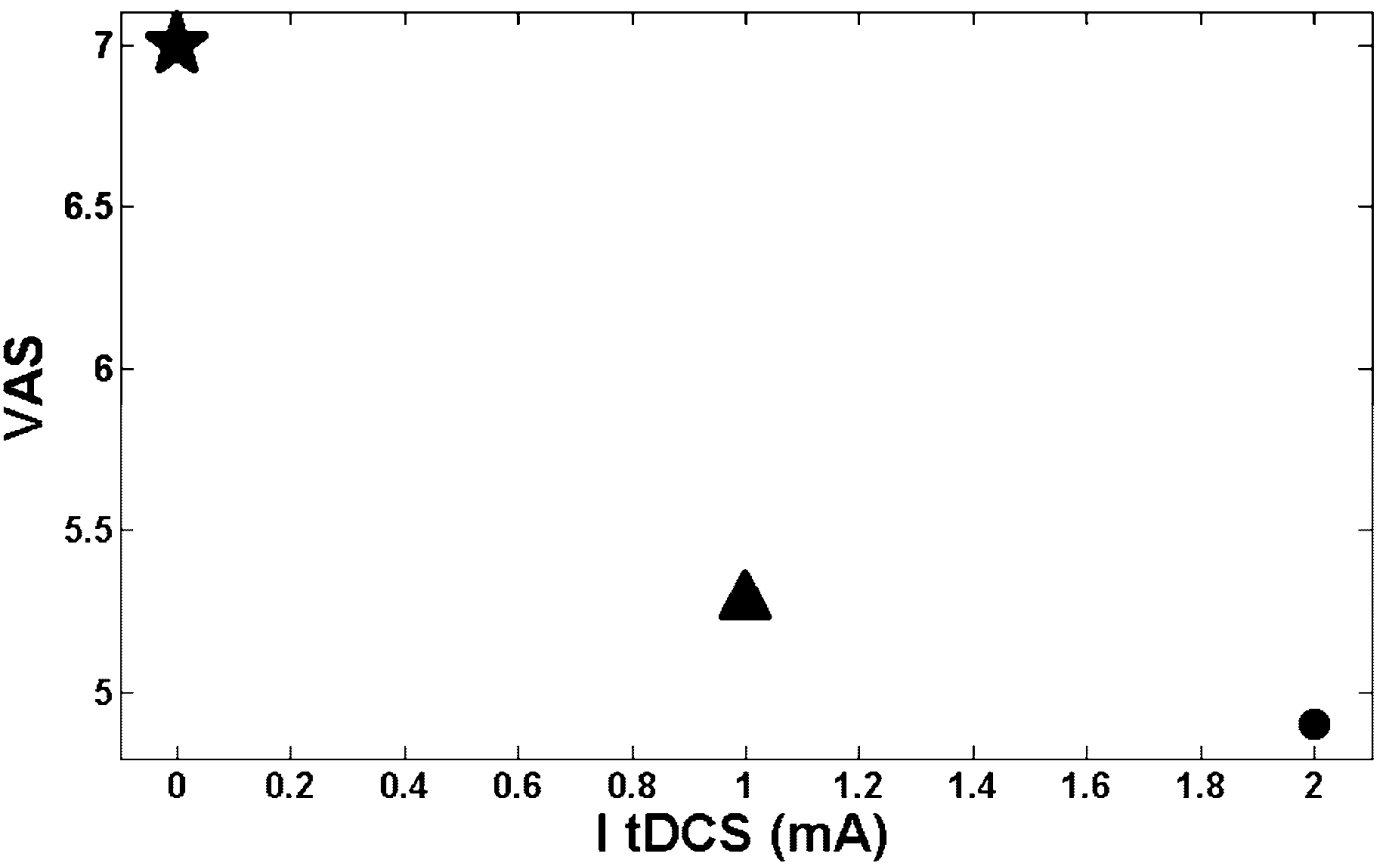
The relationship between ItDCS and the mean value of VAS. The star point shows the mean level of VAS when there is no stimulation obtained from [1, 8, 14]. The triangle point shows the mean level of VAS when $I_{tDCS}= 1\mbox{ mA}$ obtained from [1, 8, 14]. The circular point shows the mean level of VAS when $I_{tDCS}= 2\mbox{ mA}$ obtained from [15]

According to the results of the studies [1, 8, 14, 15] done on the effect of tDCS on the pain level (VAS), especially on TN, the points shown in Fig. 12 are extracted by calculating the average VAS in each current stimulation in all mentioned studies.

According to Fig. 12, increasing the level of $I_{tDCS}$ led to the decrement of the pain level (VAS) exponentially.

**Figure 13:**
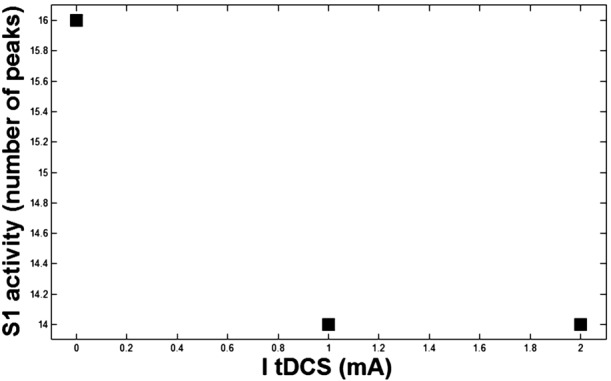
The output of model according to ItDCS

By using the simulation results of the proposed model, the relationship between the rate of S1 activity and $I_{tDCS}$ level is shown in Fig. 13.

**Figure 14:**
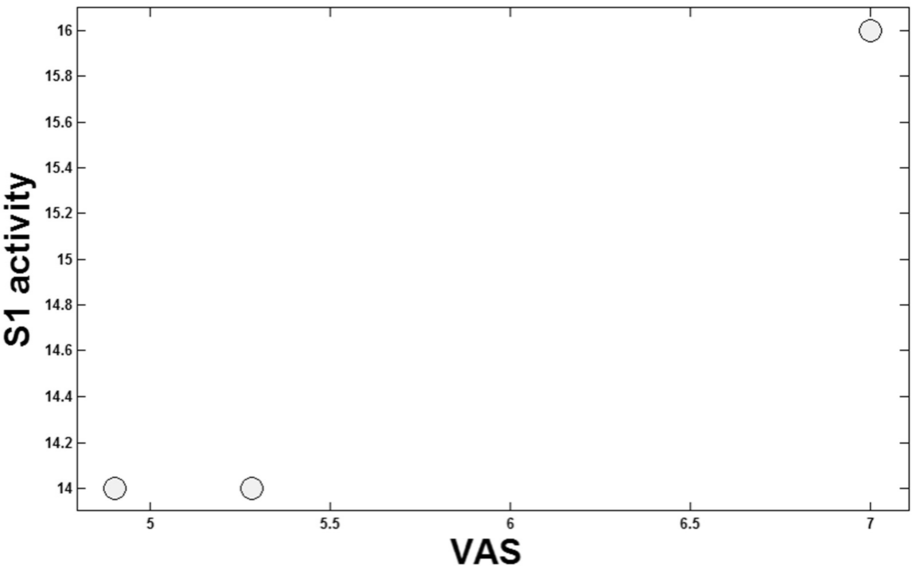
Map of S1 output on VAS

Figure 13 shows an exponential relationship between the S1 activity and the strength of $I_{tDCS}$. As discussed in the introduction part, (1) the pain level is determined by the VAS; (2) electrical stimulation ($I_{tDCS}$) affects the pain level, and (3) the TN as a neuropathic pain changes the pattern of the activities of the neurons [52]. Considering these three points and combining the results presented in Figs. 12 and 13, an exponential relationship between S1 activity and VAS index is obtained (see Fig. 14).

According to Fig. 14, increasing the S1 activity can lead to the increment of the pain level (VAS).

## Discussion

TN is one the most severe forms of pain known among other neuropathic pains but the treatment of it is still a challenge. Today, different treatment methods such as drugs, microvascular decompression, or surgeries are applied to reduce TN pain. Recently, tDCS has attracted scientific attention as a tool for pain reduction, which can be considered a safe, cheap, and accessible intervention. It has been believed that it may have influence on neurotransmitters (e.g., Glutamate or GABA) and ion concentration in intracellular and extracellular environments. For example, anodal tDCS, which is usually an excitatory stimulation, results in GABA reduction [60]. On the other hand, the cathodal stimulation, which is generally an inhibitory stimulation, causes a decrease of glutamatergic neuronal activity with a highly correlated decrease in GABA [60, 61]. Despite the mentioned suggestions, it is still unknown how tDCS affects neural processing. In this study, we aimed to provide some possible ideas about the relationship between the effects of tDCS and the associated components in TN, using a computational model. According to the results of this study, as shown in Fig. 6, the improper synthesis of proteins in particular channels (Na_v_1.8) may result in a decrease in the sensation of neuropathic pain. As mentioned, some related factors of slow sodium channels are believed to be analgesic highly selective medicines, and abnormal sensitivity to pain is removed by descending impulse activity of the Na_v_1.8 channels [62, 63]. The treatment with carbamazepine, for example, which interacts with and blocks such sodium channels, alleviates the symptoms of trigeminal neuralgia [64]. Because of the relationship between the activity of these channels and the pain, noxious mediation may cause abnormal sensitivity states like hyperalgesia, which may be treated by diminishing the activity of Na_v_1.8 [52]. The catastrophic pain of TN may stem from the activity of these sodium channels in the trigeminal ganglion. Also, pain features affect the response frequency of involved neural systems by influencing neurochemical interactions.

The proposed computational model includes some of the main regions involved in TN. Each of these regions was modeled by a modified version of the HH differential equations, and the results of the simulations demonstrated some effects of the pain stimulus and tDCS on the activity patterns of the model’s components. Although considering the population effects of a large number of neurons involved in each area of the TN pathway is highly valuable and is necessary for a better simulation, the complexity of the computations for considering an MHH for each neuron would definitely prevent us even from getting such available results. The added equations to the HH model cause it to be six differential equations which are considered for each block. Thus, it is almost impossible to have a simulation by taking a large number of MHH equations into account in this kind of modeling with Matlab software. As a result, better modeling with more similarities with the real situation should be considered as future study. It is worth mentioning that, with increasing the number of equations, the complexity of the model would be increased significantly, and the model cannot be studied and verified analytically.

Besides, based on Thévenin’s theorem, any black box containing resistances only and voltage and current sources can be replaced by a Thévenin equivalent circuit consisting of an equivalent voltage source in series connection with an equivalent resistance; thus the MHH equation can be considered as a model of the global behavior of a population of neurons in the black box (block) of each part of the brain.

According to Fig. 3, increasing the strength of the input stimulus (i.e., pain) led to the decrement of the peak to peak output potential value and the increment of the output frequency (i.e., the number of peaks). That is, as shown in Fig. 3, a further increment of the pain stimulation does not necessarily result in the increase of the S1 activity and consequently pain levels. It seems that there is a nonlinear relationship between the activity of S1 and the strength of the pain stimulus.

The trigeminal ganglion and somatosensory neuronal potential behaviors are shown in Fig. 4. While the noxious stimuli are increased, the output peak-to-peak activities of both TG and S1 blocks would be increased as well. The augmentation of the number of peaks and the amplitude of the activities was observed. Therefore, it can be speculated that the more intensifying pain sensation in S1 and TG, the more annoyance and irritation for the patients would occur.

The conductivity of pain channels, $g_{ {NaS} }$, is an influential and substantial parameter of pain propagation in the neuronal pathway [52]. The outputs of the TG and S1 regions, corresponding to the two different pain channels’ conductivity, are shown in Fig. 6. The slow sodium channels, pain channels, synthesis, and activity are reduced by the decrement of the Na_v_1.8 channels conductivity. Thereby, the transmission of pain signals may be propagated by decreasing the activity of $g_{ {NaS} }$, which may lead to pain relief. Incidentally, the amplitude variation of outputs (e.g., Fig. 6) is because of the TN input pattern fluctuation. As we can see in Fig. 6(b), the existence and absence of TN are more obvious than Fig. 6(a) with a lower conductivity of the pain channels $g_{ {NaS} }$. In other words, the different peak-to-peak values in different time steps stemmed from the existence and absence of TN.

The diagrams related to Figs. 5 and 7 show the doubling behavior of the output activity of the S1. As mentioned, they exhibit the bifurcating behavior both in the first part of the figures and then in the second part of them, which are displaying the damping period of them. In Figs. 5, 7, 8 to 11, the main point is that the range of the activity of the block is determined in a specific range, so we can consider it as a ‘*resonator*’ instead of an ‘*integrator*.’

In Figs. 8 and 9, the diagram of the nonlinear behavior of the system when changing the values of I0 and $g_{ {NaS} }$ parameters is provided. In Fig. 8, the output of the S1 block is activated in the range between two Hopf bifurcation points. Therefore, by considering the conductivity of slow sodium channels ($g_{ {NaS} }$) equal to 100, the somatosensory block would resonate in a specific range of input stimulus, which is related to a painful stimulus. Mathematically controlling the time of the occurrence of a bifurcation point is possible [65]. Using such mathematical concepts, it has been shown that controlling the neuronal behaviors is also possible [65]. In the current study, the onset of Hopf bifurcation points (shown in Fig. 8) can be controlled. That is, the interval between two Hopf bifurcation points can be changed by a specific parameter. The parameter that can control the location of the Hopf bifurcations is $g_{ {NaS} }$. By decreasing the amount of $g_{ {NaS} }$, the somatosensory block would resonate in a smaller range of input stimulus and output activity of S1, which results in a more limited range. On the other side, increasing the $g_{ {NaS} }$ will result in a higher range of input stimuli and output activity of S1, as well. In conclusion, by controlling the value of the parameter $g_{ {NaS} }$, the Hopf bifurcation points and pain related to it can be mediated.

As shown in Fig. 9, the output of the S1 block is activated in the interval between two Hopf bifurcation points. Therefore, considering the painful input stimulus equal to 30, the somatosensory block would resonate in a specific range of conductivity of slow sodium channels, which are related to pain and its pathways. The Hopf bifurcation points in Fig. 9 can be changed by modifying the *I*0 value. That is, by increasing the amount of *I*0, the Hopf bifurcation points would be close to each other and the S1 activity would be in a more limited region. By the increment of the S1 activity, the pain-related signals increase as well. Therefore, by keeping both Hopf bifurcation points far from each other, we have less pain-related signals.

In Fig. 10, the diagram shows Hopf bifurcation curves in the $(I0, g_{ {NaS} })$-plane. By increasing the values of *I*0 from negative to positive, the output values of S1 show more activity, and the number of spikes increases. As mentioned before, while we choose an *I*0 and a $g_{ {NaS} }$ from the inside of the oval-shaped figure, the output values of the S1 shows oscillation, and a periodic behavior appears, which means the increment of pain-related signals. As a result, the less oval-shaped the figure is, the less pain-related signal (i.e., periodic signal) appears in the output activity of S1.

In Fig. 11, by applying an external electrical current to the motor cortex block, we see the effect of external current stimulation. In this case, the motor cortex has two inputs. One input comes from the previous block, and another input is the electrical current stimulation over the M1 cortex. We called the second one $I_{tDCS}$. Somatosensory and motor cortices are connected functionally and structurally [6]. Therefore, they can influence each other and cause a reduction in the sensation of pain. In this regard, the essential role of the motor cortex for alleviating pain will be shown in a certain measure, and such neuronal and functional connections between M1 and other pain-related regions of the brain, especially S1, are deemed to be the most crucial part of discussing pain relief by applying external electrical stimulation. In other words, by applying anodal stimulation over the M1 area, the sub-cortical, cortico-cortical, thalamocortical and connections in the brain may be modulated, which can result in increasing the activity of significant circuits as inhibitory pain pathways, e.g., the thalamus and PAG, in the brain, which then causes pain relief. As shown in Fig. 11, by applying $I_{tDCS}$, the somatosensory cortex (S1) potential activity is reduced. Therefore, it can be suggested that the pain signals in the region of S1 will be less sensed, which is consistent with the reported results of the pain relief by tDCS [8].

Another capability of the model is its potential to map the S1 activities into the VAS value. That is, if we get the output potential activity of S1 from the model, we can approximately estimate the VAS value. Then we can find the amount of applied stimulation to have a desired amount of VAS. About the tDCS variation for the motor cortex block, it is shown in Fig. 13 that there was no difference in the model, as it shows little difference between 1 mA and 2 mA even in reality (Fig. 12), which is about 0.5 in the VAS for TN.

It is worth mentioning that considering merely three points is a bit unconvincing for contributing an exponential relation between VAS and the activity of S1. The point is that there are very small numbers of experiments in this area working on tDCS and TN. So it still needs to be validated and improved by further studies. Besides, Fig. 14 has been extracted from Figs. 12 and 13, which are obtained from the experiments and the model, respectively. So, there has been no experiment in which we could find a 0.5 mA or 1.5 mA tDCS current to apply. As a result, the plotted numbers between 0 to 1 and 1 to 2 have nothing to show as regards the results in Fig. 14, as well.

## Conclusion

The developed pain neuromatrix of TN consisted of main regions of the brain that were modeled by an MHH model. By the current version of the model, the possible effect of increasing the pain strength and also the external current stimulus on the TN neuromatrix components were investigated. For future work, other interventions (e.g., transcranial alternating current stimulation (tACS)) to other blocks of the model are suggested. The values of the conductivity and the capacitance could be specified for each block separately in a future study to have a more accurate model.

## Abbreviations

ACC: anterior cingulate cortex
HH: Hodgkin-Huxley
M1: primary motor cortex
MHH: modified Hodgkin-Huxley
PAG: periaqueductal gray
PB: parabrachial nucleus
S1: primary somatosensory cortex
S2: secondary somatosensory cortex
tACS: transcranial alternating current stimulation
tDCS: transcranial direct current stimulation
TG: trigeminal ganglion
TN: trigeminal neuralgia
VAS: visual analog scale
VPL: ventral posterolateral nucleus
VPM: ventral posteromedial nucleus
